# *Clostridium autoethanogenum* alters cofactor synthesis, redox metabolism, and lysine-acetylation in response to elevated H_2_:CO feedstock ratios for enhancing carbon capture efficiency

**DOI:** 10.1186/s13068-024-02554-w

**Published:** 2024-09-03

**Authors:** Megan E. Davin, R. Adam Thompson, Richard J. Giannone, Lucas W. Mendelson, Dana L. Carper, Madhavi Z. Martin, Michael E. Martin, Nancy L. Engle, Timothy J. Tschaplinski, Steven D. Brown, Robert L. Hettich

**Affiliations:** 1 Bredesen Center for Interdisciplinary Research, Graduate School of Genome Science and Technology, University of Tennessee, Knoxville, TN USA; 2 Oak Ridge National Laboratory, Oak Ridge, TN 37831 USA; 3 LanzaTech Inc., Skokie, IL USA

**Keywords:** *Clostridium autoethanogenum*, Multi-omics, Proteomics, Metabolomics, Acetogen, Gas fermentation, Redox, Post-translational modification, Lysine-acetylation, Clostridia

## Abstract

**Background:**

*Clostridium autoethanogenum* is an acetogenic bacterium that autotrophically converts carbon monoxide (CO) and carbon dioxide (CO_2_) gases into bioproducts and fuels via the Wood–Ljungdahl pathway (WLP). To facilitate overall carbon capture efficiency, the reaction stoichiometry requires supplementation of hydrogen at an increased ratio of H_2_:CO to maximize CO_2_ utilization; however, the molecular details and thus the ability to understand the mechanism of this supplementation are largely unknown.

**Results:**

In order to elucidate the microbial physiology and fermentation where at least 75% of the carbon in ethanol comes from CO_2_, we established controlled chemostats that facilitated a novel and high (11:1) H_2_:CO uptake ratio. We compared and contrasted proteomic and metabolomics profiles to replicate continuous stirred tank reactors (CSTRs) at the same growth rate from a lower (5:1) H_2_:CO condition where ~ 50% of the carbon in ethanol is derived from CO_2_. Our hypothesis was that major changes would be observed in the hydrogenases and/or redox-related proteins and the WLP to compensate for the elevated hydrogen feed gas. Our analyses did reveal protein abundance differences between the two conditions largely related to reduction–oxidation (redox) pathways and cofactor biosynthesis, but the changes were more minor than we would have expected. While the Wood–Ljungdahl pathway proteins remained consistent across the conditions, other post-translational regulatory processes, such as lysine-acetylation, were observed and appeared to be more important for fine-tuning this carbon metabolism pathway. Metabolomic analyses showed that the increase in H_2_:CO ratio drives the organism to higher carbon dioxide utilization resulting in lower carbon storages and accumulated fatty acid metabolite levels.

**Conclusions:**

This research delves into the intricate dynamics of carbon fixation in *C. autoethanogenum*, examining the influence of highly elevated H_2_:CO ratios on metabolic processes and product outcomes. The study underscores the significance of optimizing gas feed composition for enhanced industrial efficiency, shedding light on potential mechanisms, such as post-translational modifications (PTMs), to fine-tune enzymatic activities and improve desired product yields.

**Supplementary Information:**

The online version contains supplementary material available at 10.1186/s13068-024-02554-w.

## Background

The Intergovernmental Panel on Climate Change (IPCC) has recently stated that limiting human-caused global warming requires net-zero CO_2_ emissions, and our collective actions to limit greenhouse gas emissions in the next decade will determine whether warming can be limited to 1.5–2.0 °C above the 1850–1900 average [1]. Yet, an intensified demand for energy, fuel, and chemicals continues to drive emissions higher year after year and, despite a brief reduction due to COVID-19 lockdowns, global energy-related CO_2_ emissions rose to an all-time high of 37 Gt in 2022 [2]. While dire, the situation is not hopeless. Multiple pathways exist for decarbonizing the energy sector, which will become more incentivized with appropriate policy implementations [3, 4]. However, the demand for chemicals, materials, and energy-dense fuels for maritime shipping or air travel is expected to continue to increase and will be dependent on fossil resources for an extended time. Pivoting from finite, fossil-based resources to a biology-based circular carbon economy will require microbial fermentation at very large scales to enable sufficient sustainable manufacturing [5]. Though fermentation has been conducted at an industrial scale using sugars/starch for over a century [5, 6], the process has inherent economic challenges, along with competition from low-cost fossil alternatives that have stymied its implementation at commercial scales. More so, traditional fermentation from sugars to ethanol is carbon inefficient, as one mole of CO_2_ is produced for every mole of ethanol [7].

**Fig. 1:**
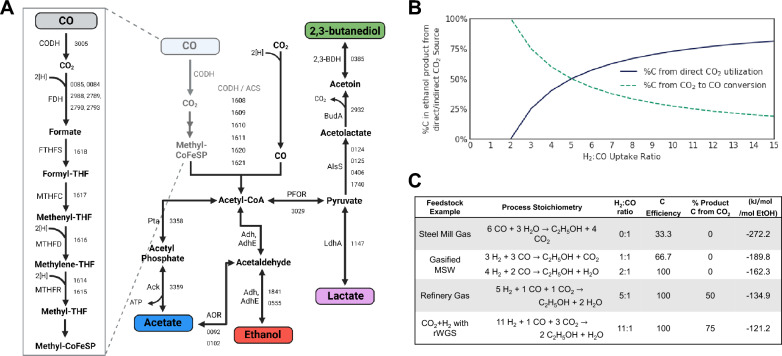
Minimizing CO as a feedstock will increase direct CO_2_ consumption by *C. autoethanogenum*. (**a**) The Wood–Ljungdahl pathway is the metabolic pathway that *C. autoethanogenum* uses to convert CO and CO_2_ into ethanol and acetate. Truncated locus tags are provided with “CAETHG_” identifier removed. **b** A chart showing that as the H_2_:CO uptake ratio of chemostats increases, direct CO_2_ consumption increases with concomitant and inverse decrease of indirect CO_2_ consumption. The solid blue line is the percent of carbon that is being consumed directly from CO_2_. The dotted green line represents the percent of carbon that is being taken up as CO_2_ then converted to CO. **c** The reaction stoichiometry of ethanol production under various gas feedstocks. The increase in overall carbon conversion, increase in direct CO_2_ into product, and decrease in run length are all shown as a result in the stoichiometric ratios increasing H_2_:CO uptake ratios. Free energy change of reactions were calculated with eQuilibrator. CODH: CO dehydrogenase; FDH: formate dehydrogenase; FTHFS: formyltetrahydrofolate synthetase; MTHFC: Methenyltetrahydrofolate cyclohydrolase; MTHFD: Methenyltetrahydrofolate dehydrogenase; MTHFR: Methylenetetrahydrofolate reductase; Pta: phosphotransacetylase; Ack: acetatekinase; CODH/ACS acetyl-CoA synthase/carbon monoxide dehydrogenase complex; Adh/AdhE: Alcohol Dehydrogenase; AOR: acetaldehyde:ferredoxin oxidoreductase; LdhA: Lactate Dehydrogenase; AlsS: Acetolactate synthase catabolic; BudA: acetolactate decarboxylase; 2,3-BDH: 2,3-Butanediol Dehydrogenase; MSW: Municipal solid waste; rWGS: reverse water–gas shift

Gas fermentation has emerged as a promising addition to the portfolio of technologies required to realize a sustainable circular economy [8]. In this process, a gaseous one-carbon substrate (e.g., carbon monoxide, carbon dioxide, methane) is assimilated into the biomass of chemolithoautotrophic microorganisms and fermented to additional products. One prominent group of gas-fermenting microorganisms are the obligately anaerobic acetogens [9], who can fix CO_2_ via the Wood–Ljungdahl pathway (WLP) (Fig. 1a), the most-efficient known biological carbon fixation pathway [10] and arguably the oldest overall biological pathway [11]. The WLP, which was elucidated over decades, proceeds through two branches [12]: (i) the “eastern” or methyl- branch where CO_2_ is reduced to formate, affixed to tetrahydrofolate, and further reduced to methyl-tetrahydrofolate before the methyl group is moved to a cobalamin-containing transferase protein, and (ii) the “western” or carbonyl- branch in which CO_2_ is reduced to carbon monoxide (CO) (or CO is utilized directly). The branches meet at the acetyl-CoA synthase/carbon monoxide dehydrogenase complex (ACS/CODH), which combines the methyl group with the CO to form a molecule of acetyl-CoA. This critical pathway has been found across many phylogenetic classes and has been observed to operate in the oxidative and reductive directions based on the metabolic context of its host [9].

LanzaTech has commercialized an ethanol-producing gas fermentation process with *Clostridium autoethanogenum*, a WLP-utilizing acetogen, using industrial gaseous waste feedstocks from steel mills, ferroalloy plants and petrochemical refineries [13]. Steel-mill feedstocks typically have a high CO composition, which can provide both carbon and electrons to fuel microbial growth, but the process is also carbon inefficient due to the production of CO_2_ from CO [14]. The stoichiometry of introducing a greater proportion of H_2_ in the feed gas leads to a quantitative increase in carbon efficiency to the extent that H_2_:CO uptake ratios > 2:1 are sufficient to achieve 100% carbon efficiency (Fig. 1b, c). More specifically, as the H_2_:CO uptake ratio increases, CO_2_ can be directly utilized as a carbon source and fixed into fermentation products such as ethanol; thus, fermentation of gas streams with increasing amounts of H_2_ allows for the direct incorporation of CO_2_ into valuable products to approach a true carbon-negative production scheme. This enables the mitigation of carbon emissions from a broader diversity of commercial-scale feedstocks, such as CO_2_-rich point sources like first-generation corn ethanol plant tail gas and direct-air CO_2_ capture.

While high H_2_:CO ratios appear stoichiometrically attractive, in practice, they represent a daunting challenge for commercial gas fermentations producing reduced products such as ethanol. A major hurdle is the physical properties of the electron sources CO and H_2_, with CO containing more energy per mole, and the fermentation of CO to ethanol being significantly more thermodynamically favorable than CO_2_ + H_2_ (Fig. 1C) [15, 16]. Optimizing *C. autoethanogenum* to use CO_2_ as its primary carbon source is essential to improve its impact on climate change mitigation and increase its carbon recycling potential. Earlier studies have compared steady-state CO_2_/H_2_ (~ 23% CO_2_, ∼ 67% H_2_) to fermentations supplemented with CO (CO, ∼ 23% CO_2_, ∼ 65% H_2_) and modeled the different fermentations, while others have demonstrated that increasing available hydrogen impacts product distribution in a comparison of elevated CO (~ 60% CO) and hydrogen conditions (~ 15% CO, 45% H_2_) [17, 18]. 

To better quantify and understand the process outlined above, we examined growth of *C. autoethanogenum* in chemostats with two different H_2_:CO target uptake ratios (5:1 H_2_:CO and 11:1 H_2_:CO), hypothesizing that a correlative relationship will emerge between decreased CO consumption and an increased capacity for direct CO_2_ conversion. Note that this ultra-high H_2_:CO ratio (11:1), which exhibits 100% carbon efficiency and 75% direct CO_2_ fixation to ethanol, is a highly desirable condition but has been untested as to its effect on metabolic activity, redox properties, and stability of the cultured bacterium. We then conducted multi-omics and post-translational modification (PTM) analyses on chemostat samples to investigate the molecular changes in the proteome and metabolome, with a particular focus on the WLP and C1 metabolism-related pathways. As molecular hydrogen is the key difference in these systems, detailed focus was given to examination of hydrogenases in the two conditions. This study confirms previously published results looking at shifts in *C. autoethanogenum* H_2_:CO uptake and correlated protein expression [17–20], but ventures further by providing *C. autoethanogenum* post-translational modification (PTM) profiles and intracellular metabolomic analyses by GC–MS. The global profiles and insights will guide future cellular engineering efforts to increase the efficiency of *C. autoethanogenum* in utilizing CO_2_ as its primary carbon source.

## Results

### H_2_:CO ratio changes cause CO uptake level shifts in steady-state chemostats

Stoichiometric calculations show that the addition of hydrogen and the minimization of CO in a feedstock increases direct CO_2_ conversion (Fig. 1b). An increase in the H_2_:CO uptake ratio from 2:1 up to 11:1 will increase the direct incorporation of CO_2_ into product carbon to 75% (Fig. 1c). We conducted continuous stirred tank reactor (CSTR) experiments to assess the effect of feedstock on phenotype. The cultures were maintained in pH-controlled chemostats using two distinct gas compositions: a “low H_2_:CO ratio” (50% H_2_ and 5% CO) and a “high H_2_:CO ratio” (60% H_2_ and 3% CO). We selected these conditions to target a 5:1 and 11:1 H_2_:CO uptake ratio and achieve 50% and 75% direct CO_2_ fixation to ethanol, respectively (Fig. 1c).

**Fig. 2:**
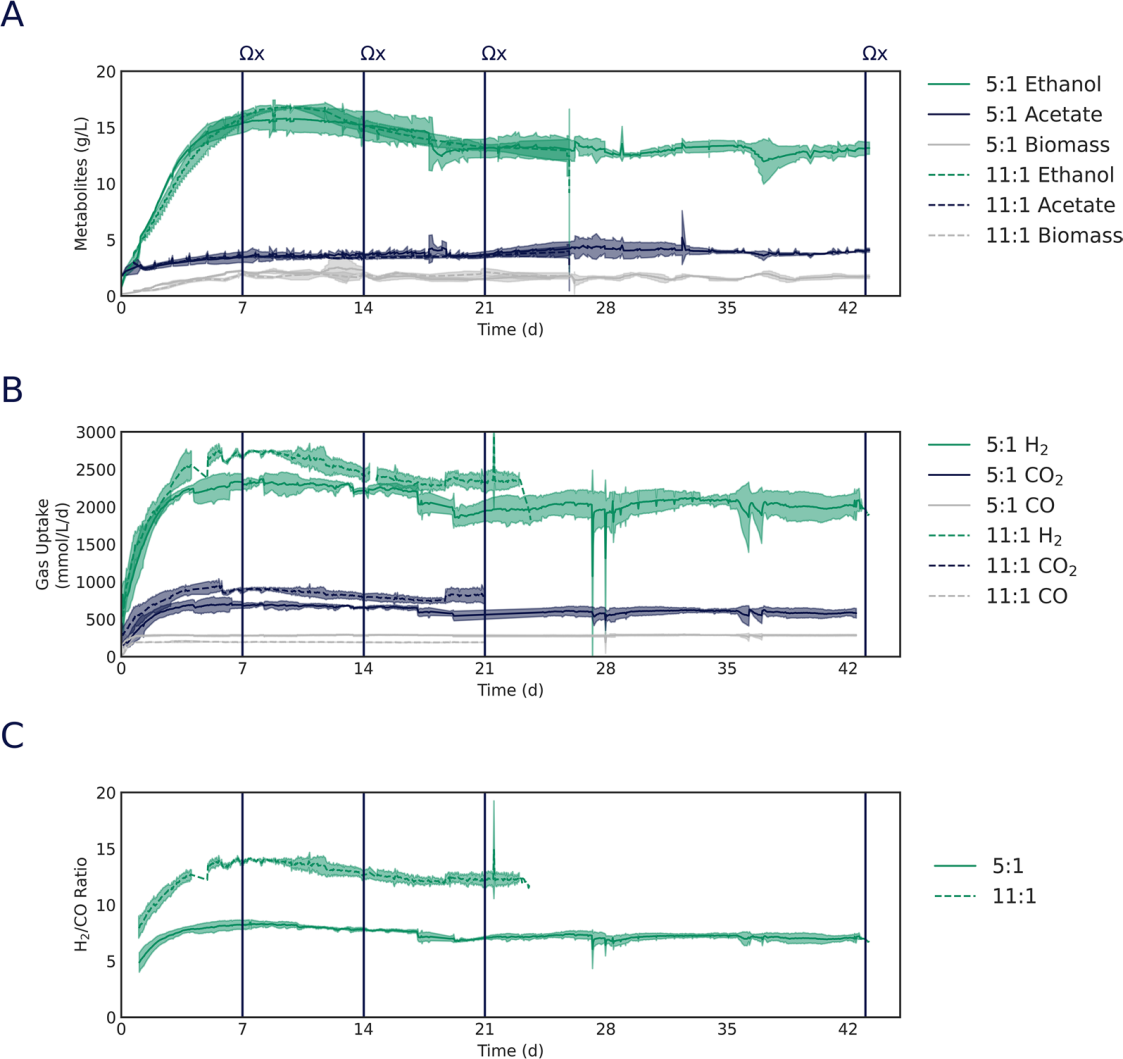
Change in H_2_:CO ratio shifts chemostats gas uptake profiles while maintaining steady end-product formation. Comparison of major metabolites (**A**), molar gas uptake rates (**B**), and observed H_2_:CO uptake ratios (**C**) during triplicate fermentations conducted under 5:1 and 11:1 target uptake condition. Vertical lines indicate when -omics samples were obtained for 5:1 target uptake (solid) and 11:1 target uptake (dashed) conditions

Our results indicate that despite the variations in gas mixture composition, the steady-state biomass concentrations were consistent at ~ 2 g/L and titers of the main metabolites were similar (Fig. 2a, Table 1). Furthermore, we observed differences in the gas uptake rates between the two conditions (Fig. 2b, c). In the low H_2_:CO ratio condition, over days 7–21, the average CO uptake rate was 278.2 mmol/L/d, with an average H_2_:CO uptake ratio of 7.8 and a H_2_:CO_2_ uptake ratio of 3.2. This confirms a higher consumption of CO compared to the high H_2_:CO ratio condition, as expected. In contrast, the high H_2_:CO ratio condition displayed an average CO uptake of 169.6 mmol/L/d, with an average H_2_:CO ratio of 13.1 and a H_2_:CO_2_ ratio of 3.1 (Table 1). These results demonstrate that similar titer and productivity can be maintained while incorporating more CO_2_ directly into the desired product by shifting the H_2_:CO ratio.

**Table 1:** Fermentation performance metrics under different H_2_:CO ratios

		5:1 (d7-d21)		11:1 (d7-d21)	
		Mean	Std Dev	Mean	Std Dev
Titer					
Ethanol	g/L	14.9	1.6	15.5	1.5
Acetate	g/L	3.7	0.4	3.5	0.2
Biomass	gDCW/L	1.9	0.5	1.8	0.2
Operation					
D	1/d	0.89	0.06	0.76	0.06
Db	1/d	0.16	0.04	0.11	0.05
Productivity					
Ethanol	g/L/d	13.3	1.7	11.7	1.3
Acetate	g/L/d	3.3	0.4	2.6	0.3
Selectivity					
Ethanol	C %	83.1	2.0	84.7	2.2
Acetate	C %	15.2	2.1	13.8	0.9
Biomass	C %	1.6	0.4	1.2	0.4
Carbon balance		99.9		99.8	
Uptake rate					
CO	mmol/L/d	278.2	38.3	169.6	7.7
H_2_	mmol/L/d	2163.6	395.8	2223.6	155.8
CO_2_	mmol/L/d	682.6	260.4	725.8	84.3
Uptake ratio					
H_2_:CO		7.8		13.1	
H_2_:CO_2_		3.2		3.1	

gDCW: grams dry cell weight; D: dilution rate; Db: bacterial dilution rate. Data are the mean ± standard error of the mean of 3 replicate bioreactors

### The overall proteome shows specific protein abundance shifts as a function of H_2_:CO ratios

Proteomic measurements were conducted for multiple time points across steady states for each chemostat condition and yielded detailed molecular information for each sample, as shown in Additional file 1: Table S1-5. Based on the overall protein abundance distributions, the timepoints within each condition clustered tightly on a PCA plot and separated only by condition, with one notable exception (Additional File 2: Figure S1).

Of the three timepoints taken from the high H_2_:CO condition, the day 7 timepoint samples were distinct. This condition had the highest number of unique proteins and contributed to the vast majority of differentially expressed proteins within the conditions across the time series (Additional File 3: Table S6). In general, almost all proteins, including the central WLP and hydrogenases, followed a trending decrease in abundance from day 7 to day 21 (Additional File 3: Table S7-8), with consistent abundance from day 14 to 21 (Additional File 3: Table S9). Upon closer inspection, it appears that the day 7 sample had not yet reached steady-state conditions for the proteome, so it was not included in any further detailed proteome analysis.

Since the latter time points for each chemostat exhibited similar proteomic profiles, the data from each time point set, i.e., the low H_2_:CO condition days 20 and 43 (*n* = 6), and the high H_2_:CO ratio days 14 and 21 (*n* = 6), were grouped together to compare the proteomic differences between the two conditions (Additional File 4: Table S10).

The time-pooled proteomic measurements revealed a comparable number of proteins in both conditions at steady state; 1827 proteins were identified in the low H_2_:CO ratio condition and 1831 in the high H_2_:CO ratio condition (Additional File 4: Table S10). Upon initial inspection of protein rank order by abundance, in both conditions, the top-most-abundant proteins were consistent even though relative ordering of these varied. Within the top 15 most-abundant proteins are 9 that are directly involved in the WLP (out of the 33 total possible WLP proteins), verifying the importance and consistency of the WLP (Additional File 4: Table S10). A detailed comparison of the complete measured proteome between the two gas ratios revealed 515 significantly changing proteins (*p* value < 0.05), with 226 of these exhibiting a log2 fold-change (FC) greater than one.

Based on the information listed above and as an obvious starting point, a detailed examination of the proteins most differentially changed as a function of the hydrogen ratio was examined. The top 50 rank-ordered differentially expressed proteins in the comparison between the low and high H_2_:CO conditions were populated by a variety of unrelated proteins: some transporters, a few ribosomal proteins, a protein involved in citrate degradation step of TCA cycle, cobalamin B12 binding domain protein, and a dehydrorhamnose reductase protein (Additional File 4: Table S10). After extensive examination of the most significant changing proteins, no obvious metabolic connections to the variation of hydrogen feed gas were found; further detailed examination and testing of these somewhat unrelated proteins was beyond the scope of this project.

### Wood–Ljungdahl pathway and fermentation end products

Since the WLP is the core metabolism route connecting the C1 feedstock to the desired end products, we began the more detailed proteome exploration by investigating this pathway. Specifically, the WLP enzymes metabolize CO and CO_2_ into acetyl-CoA, which is then converted into ethanol, acetate, 2,3-butanediol, and/or lactate via end-product fermentation (Fig. 1a). When comparing WLP proteins across the two conditions, these proteins are some of the most abundant and conserved with regards to their expression; only 2 proteins (alpha-acetolactate decarboxylase, CAETHG_2932, and a CO dehydrogenase (CODH) subunit, CAETHG_1620, showed significant differential expression (*p* value < 0.05, and |log2 FC|> 1). CAETHG_1620 was more abundant (FC ~ 1.5) in the high H_2_:CO condition (Table 2). CAETHG_2932, a 2,3-butanediol synthesis protein (Table 2) on the other hand, exhibited a higher abundance in the low H_2_:CO condition; the potential cause for this change in abundance is not apparent as only trace amounts of both 2,3-butanediol and lactate were observed among the major fermentation products in either condition. The major fermentation products observed were acetic acid and ethanol, and no related synthesis proteins showed statistically significant differential expression. Given steady chemostat cultivation and minimal change in fermentation product profiles across conditions, this lack of significant change in enzyme abundance is not unexpected. The high abundance and modest change in the WLP proteins overall suggest that *C. autoethanogenum* does not have to drastically adjust the expression of WLP proteins to accommodate these increasingly hydrogen-rich gas streams. This is consistent with the proteomic results from related lower hydrogen ratio studies with *C. autoethanogenum*, and the similar organism, *C. ljungdahlii*, [17, 21].

**Table 2:** H_2_:CO gas uptake ratio changes induce shifts in key metabolic pathways

Protein ID	Description	*p* value	Log2 fold change
Wood–Ljungdahl pathway/fermentation end products			
CAETHG_1608	CO-methylating acetyl-CoA synthase	0.268	− 0.08
CAETHG_1609	Dihydropteroate synthase DHPS	0.321	0.08
CAETHG_1610	CO dehydrogenase/acetyl-CoA synthase delta subunit, TIM barrel	0.136	− 0.15
CAETHG_1611	CO dehydrogenase/acetyl-CoA synthase delta subunit, TIM barrel	0.018*	0.29
CAETHG_1614	Methylenetetrahydrofolate reductase	0.139	0.15
CAETHG_1615	Methylenetetrahydrofolate reductase domain-containing protein	0.226	− 0.31
CAETHG_1616	Bifunctional protein FolD	0.128	0.18
CAETHG_1617	Methenyltetrahydrofolate cyclohydrolase	0.794	0.04
CAETHG_1618	Formate–tetrahydrofolate ligase	0.362	− 0.11
CAETHG_1620	Carbon monoxide dehydrogenase (Acceptor)	0.002*	− **1.50**
CAETHG_1621	Carbon monoxide dehydrogenase (Acceptor)	0.994	0.00
CAETHG_2789	Molybdopterin oxidoreductase	0.233	− 1.88
CAETHG_2790	Formate dehydrogenase, alpha subunit	0.107	0.27
CAETHG_2793	Protein FdhD	0.080	− 0.21
CAETHG_3005	Carbon monoxide dehydrogenase	0.006*	− 0.38
CAETHG_3899	Carbon monoxide dehydrogenase	NF	NF
CAETHG_0084	Nitrate reductase	0.648	− 0.04
CAETHG_0085	Nitrate reductase	0.241	0.17
CAETHG_2988	Formate dehydrogenase, alpha subunit	0.045*	− 0.42
CAETHG_0092	Aldehyde ferredoxin oxidoreductase	0.004*	-0.44
CAETHG_0102	Aldehyde ferredoxin oxidoreductase	0.884	− 0.06
CAETHG_3358	phosphate acetyltransferase	0.004*	0.49
CAETHG_3359	Acetate kinase	0.934	0.01
CAETHG_0555	Alcohol dehydrogenase	0.983	− 0.01
CAETHG_1841	Alcohol dehydrogenase	0.062	− 0.41
CAETHG_3029	Pyruvate ferredoxin/flavodoxin oxidoreductase	0.031	0.44
CAETHG_0124	Acetolactate synthase	0.900	0.02
CAETHG_0125	Acetolactate synthase small subunit	0.087	− 0.26
CAETHG_0406	Acetolactate synthase	0.006	− 0.30
CAETHG_1740	Acetolactate synthase	0.479	0.14
CAETHG_2932	Alpha-acetolactate decarboxylase	0.002*	**1.07**
CAETHG_0385	(R,R)-Butanediol dehydrogenase	0.748	− 0.11
CAETHG_1147	D-Lactate dehydrogenase	0.040*	0.19
B12 Biosynthesis			
CAETHG_1110	Cob(I)alamin adenosyltransferase	0.005*	0.67
CAETHG_1111	GHMP kinase	0.002*	**3.56**
CAETHG_1112	Precorrin-6 × reductase	0.004*	0.79
CAETHG_1113	Sirohydrochlorin cobaltochelatase	0.023*	0.77
CAETHG_1114	Precorrin-3B C17-methyltransferase	0.256	− 0.50
CAETHG_1115	Cobalamin synthesis G domain-containing protein	0.028*	**3.50**
CAETHG_1116	Precorrin-4 C11-methyltransferase	0.012*	0.47
CAETHG_1117	Cobalt-precorrin-2 C(20)-methyltransferase	0.018*	0.58
CAETHG_1118	Precorrin-6Y C5,15-methyltransferase (Decarboxylating), CbiT subunit	0.069	0.36
CAETHG_1119	Precorrin-6y C5,15-methyltransferase (Decarboxylating), CbiE subunit	0.057	0.42
CAETHG_1120	Cobalt-precorrin-5B C(1)-methyltransferase	0.039*	0.53
CAETHG_1121	Precorrin-8X methylmutase	0.009*	0.63
CAETHG_1122	Nicotinate-nucleotide–dimethylbenzimidazole phosphoribosyltransferase	0.068	0.34
CAETHG_1123	Cobyrinate a,c-diamide synthase	0.010*	0.61
CAETHG_1124	Delta-aminolevulinic acid dehydratase	0.023*	0.64
CAETHG_1125	uroporphyrinogen-III C-methyltransferase	0.068	0.37
CAETHG_1126	Porphobilinogen deaminase	0.002*	**1.56**
CAETHG_1127	Precorrin-2 dehydrogenase	0.002*	**1.03**
CAETHG_1128	Threonine-phosphate decarboxylase	0.005*	**1.74**
CAETHG_1460	Adenosylcobinamide kinase	0.107	0.22
CAETHG_1461	Adenosylcobinamide-GDP ribazoletransferase	NF	NF
CAETHG_1462	Alpha-ribazole phosphatase	0.095	0.34
Energy/redox			
CAETHG_3227	Ion-translocating oxidoreductase complex subunit C	0.066	− 0.35
CAETHG_3228	Ion-translocating oxidoreductase complex subunit D	0.607	− 0.11
CAETHG_3229	Ion-translocating oxidoreductase complex subunit G	0.137	− 0.25
CAETHG_3230	Ion-translocating oxidoreductase complex subunit E	0.509	1.76
CAETHG_3231	Ion-translocating oxidoreductase complex subunit A	NF	NF
CAETHG_3232	Ion-translocating oxidoreductase complex subunit B	0.003*	− **1.40**
CAETHG_2233	Alpha-L-arabinofuranosidase domain protein	0.120	− 2.95
CAETHG_2342	ATP synthase subunit I	NF	NF
CAETHG_2343	ATP synthase subunit a	0.092	0.26
CAETHG_2344	ATP synthase subunit c	NF	NF
CAETHG_2345	ATP synthase subunit b	0.021*	0.15
CAETHG_2346	ATP synthase subunit delta	0.628	0.38
CAETHG_2347	ATP synthase subunit alpha	0.619	0.02
CAETHG_2348	ATP synthase gamma chain	0.069	0.16
CAETHG_2349	ATP synthase subunit beta	0.043*	0.09
CAETHG_2350	ATP synthase epsilon chain	0.047*	0.14
CAETHG_1580	Glutamate synthase (NADPH), homotetrameric	0.634	0.06
CAETHG_1581	Redox-sensing transcriptional repressor Rex	0.625	− 0.04
CAETHG_2794	Electron-bifurcating FeFe-hydrogenase dependent on TPN subunit C	0.468	− 0.13
CAETHG_2795	Electron-bifurcating FeFe-hydrogenase dependent on TPN subunit B	0.629	− 0.07
CAETHG_2796	4Fe-4S ferredoxin, iron-sulfur binding domain-containing protein	0.002*	− 0.65
CAETHG_2797	4Fe-4S ferredoxin, iron-sulfur binding domain-containing protein	0.005*	0.92
CAETHG_2798	Hydrogenase, Fe-only	0.958	− 0.01
CAETHG_2799	4Fe-4S ferredoxin, iron-sulfur binding domain-containing protein	0.793	− 0.05
CAETHG_3841	Hydrogenase, Fe-only	0.146	3.01
CAETHG_0110	Hydrogenase large subunit domain protein	NF	NF
CAETHG_0860	Hydrogenase maturation protease	NF	NF
Transacetylase/deacetylase			
CAETHG_2239	NAD-dependent protein deacetylase	0.941	− 0.01
CAETHG_2992	GCN5-related N-acetyltransferase	0.007*	**− 2.59**

A table of proteins organized by pathway, or protein group. The first column depicts Locus IDs, the second column Uniprot annotations, third column are *p* values, and fourth column fold changes from comparison between the low and high H_2_:CO conditions. *P* values < 0.05 are marked by an asterisk. Proteins with significant differential expression (*p* value < 0.05 and log2 changes >|1|) are bolded. Positive fold change values indicate a higher protein abundance in the low H_2_:CO condition, and a negative fold change value indicates a higher protein abundance in the high H_2_:CO condition. NF in the Log2 fold change column stands for not found

### Vitamin B12 biosynthesis

Beyond its commonly known function in methionine synthase and as a co-factor in other enzymes, vitamin B12 is a critical cofactor in the WLP, as it facilitates the transfer of a methyl group from tetrahydrofolate to the nickel-containing A-cluster of the ACS/CODH complex for incorporation into acetyl-CoA [9]. Between both conditions, 21 of the 22 proteins in the B12 synthesis pathway were detected in the proteome measurements. Comparing the conditions revealed that 5 of the 21 detected proteins in the B12 biosynthesis pathway were significantly more abundant in the low H_2_:CO condition (Table 2). In addition, with the exception of one protein, all the identified proteins belonging to this pathway follow the same trend, showing higher expression in the low H_2_:CO condition. Nine of these proteins passed the *p* value threshold but did not have a |fold change| greater than one (Table 2). The consistency of expression levels for the entire pathway across the different conditions suggests *C. autoethanogenum* might be regulating flux through the WLP by altering the availability of B12 and this emerges as lower cell-specific carbon uptake under the high H_2_:CO condition. Conversely, the biosynthesis of B12 requires high amounts of ATP and so the cultures may be downregulating the pathway as a result of the lower energy availability (i.e., greater H_2_:CO ratio) in the feed gas.

### Hydrogen utilization and energy metabolism

Considering that the largest variable in these studies is the amount of hydrogen substrate, the proteins related to hydrogen utilization/conversion were an obvious target for analysis. *C. autoethanogenum* contains five distinct hydrogenase genes/gene clusters [22], and a mix of responses was observed across the gas blends queried in this study. Of the redox proteins related to the WLP (including those belonging to the hydrogenase gene clusters), only one, CAETHG_3232, Ion-translocating oxidoreductase complex subunit B, has significantly higher expression in the high H_2_:CO condition (Table 2). This protein is a part of the membrane-associated and energy-conserving reduced ferredoxin:NAD + oxidoreductase complex, RNF, made of six proteins, five of which were detected and were more abundant in the high H_2_:CO condition. The remaining complex proteins were not statistically significant but 3 out of 4 follow a trend similar to CAETHG_3232’s expression (Table 2). Counter to this, the ATP synthase complex seems to trend in the opposite direction. Though none of the eight out of nine identified ATP synthase complex proteins pass the commonly used threshold of a fold change greater than one log2 unit, i.e., a doubling, three proteins pass the *p *value threshold, and all have fold changes that indicate higher expression in the low H_2_:CO condition (Table 2). These results suggest minimal proteome abundance changes are needed for *C. autoethanogenum* to accommodate a higher percentage of hydrogen utilization and are in accord with a recent study comparing proteome changes between syngas (2:5 H_2_:CO) and a H_2_ + CO blend (3:1 H_2_:CO), where the redox enzyme changes were minimal [23].

### Regulatory PTMs, in particular lysine-acetylation, are connected to the increase in the H_2_:CO ratio

For the major enzymatic pathways discussed herein, our proteome measurements revealed that their protein abundances in general were largely unimpacted by the switch to the higher H_2_ condition. This suggests that additional levels of regulation may be used to control the activity of these highly abundant proteins. Protein post-translational modifications (PTMs) add an additional layer of regulation that can often go unnoticed unless specifically examined. For a deeper look into levels of protein regulation beyond simple abundance changes, we sought to examine protein post-translational modifications, specifically acetylation and biotinylation as they have been recently shown to affect autotrophic growth in *C. ljungdahlii* [24–26]. We also looked at methylation due to the prominence of C1 metabolism in WLP-containing acetogens.

### Lysine-acetylation

Lysine-acetylation has been confirmed as a post-translational modification regulatory factor in several enzymes related to central carbon metabolism for the closely related organism *C. ljungdahlii* [24, 25]. The acetyltransferase CAETHG_2992 is an orthologue of the acetyltransferase At2 of *C. ljungdahlii,* which has been shown to affect the acetylation and fermentation profiles of that organism by modulating the activity of key enzymes such as formate dehydrogenase (FDH), PTA, ADH, and Rex [24, 25] (Table 2). In this study, the presence of the transacetylase was detected at significantly greater amounts in high H_2_:CO target uptake condition and so we investigated the acetylation profile across specific metabolic pathways.

**Fig. 3:**
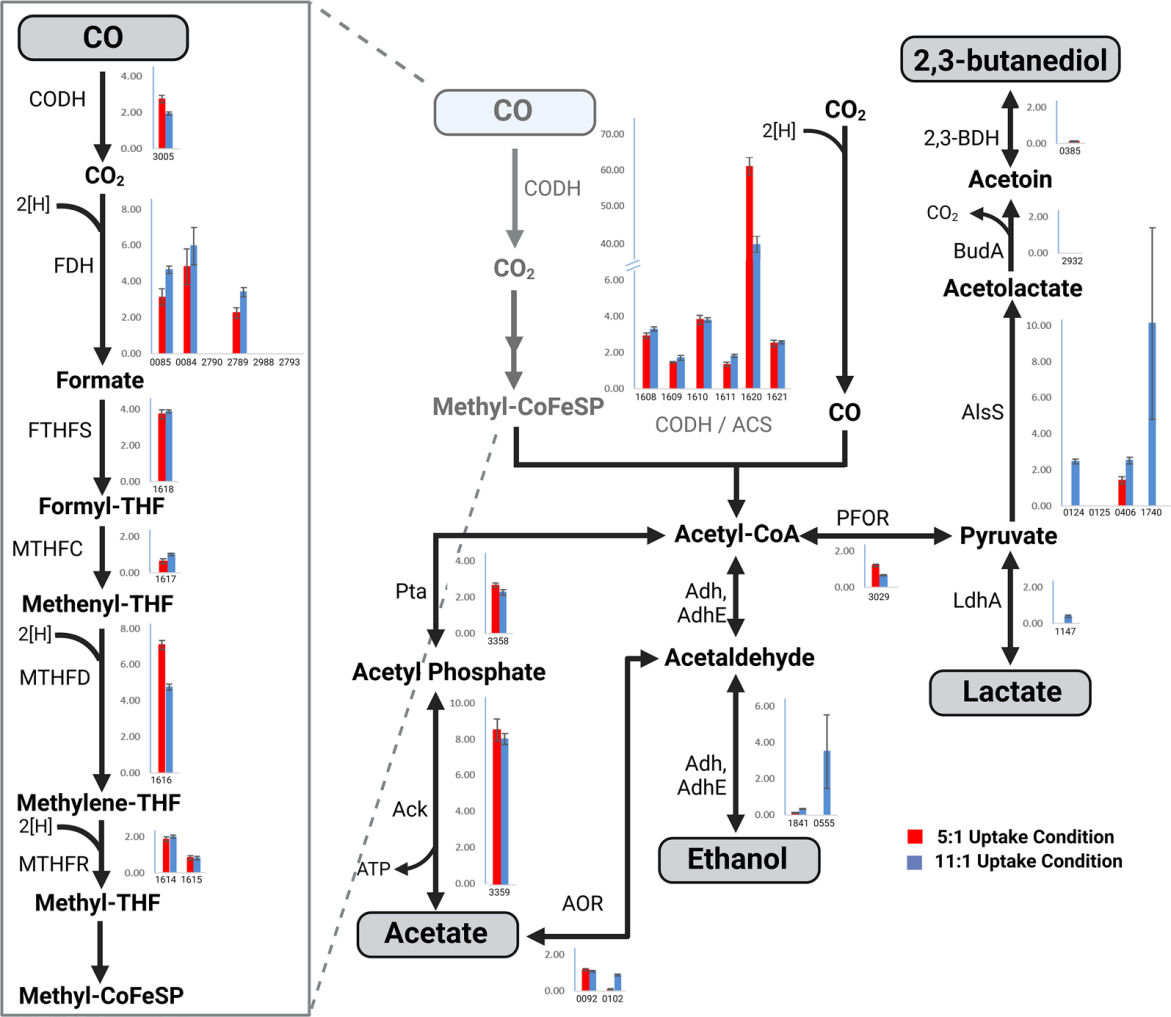
Lysine-acetylation in *Clostridium autoethanogenum* was observed in unusually high percentages A diagram of the WLP with percent abundance of acetylated peptides comparing the high and low H_2_:CO conditions. The low H_2_:CO condition is represented with red, and the high H_2_:CO condition in blue. Y-axis for each bar graph represents the percentage of acetylated peptide abundance to total proteome abundances.

Among all samples, an average of 1.9% of the total protein abundance showed evidence of lysine-acetylation (Additional File 2: Figure S2) with somewhat variable differences observed across the proteomes of each condition (Additional File 5: Table S11). Though global differences in acetylation were generally unchanged or inconsistent across the time points, this regulatory PTM is often concentrated to specific pathways or cellular sub-systems—information that would otherwise be washed out when only looking at global patterns. When specifically focusing on the WLP, the high H_2_:CO condition showed a pattern of higher acetylation in the formate dehydrogenase proteins (Fig. 3), a known target of acetylation-based post-translational regulation [25]. These proteins aid in the conversion of CO_2_ to formate, the first step in the WLP.

Notably, the alcohol dehydrogenases showed an increase in acetylation during the high H_2_:CO target uptake condition. Several proteins related to pyruvate metabolism also exhibited increased acetylation in the high H_2_:CO condition (e.g., AlsS and LdhA). Conversely, higher levels of acetylation were observed in proteins participating in the conversion of acetyl-CoA to pyruvate and methenyl-tetrahydrofolate metabolism in the low H_2_:CO condition (Fig. 3). The observation of variable acetylation suggests that enzyme activity may be regulated post-translationally to fine-tune C1 metabolism, but further work is needed to understand this in more detail. It is worth noting that the acetylation patterns were not uniform nor linked to protein abundances, indicating that this modification is not due to abiotic acetylation but rather deliberate post-translational control.

### Methylation

Protein methylation often shifts the hydrophobicity of a protein, thereby altering its structure and potentially regulating function [27]. In total, 133 proteins showed evidence of arginine and/or lysine methylation in at least one condition (Additional File 5: Table S12). Of these, 21 have methylation within at least two of the three replicates. Across conditions and time points, the percent of methylation to the total proteome abundance averaged at 0.23% and changes in global methylation abundance had negligible variation (Additional File 2: Figure S3). In these conditions, most of the methylation was attributed to lysine methylation compared to arginine, which only accounted for an average of 0.04% of the methylation across the conditions.

**Fig. 4:**
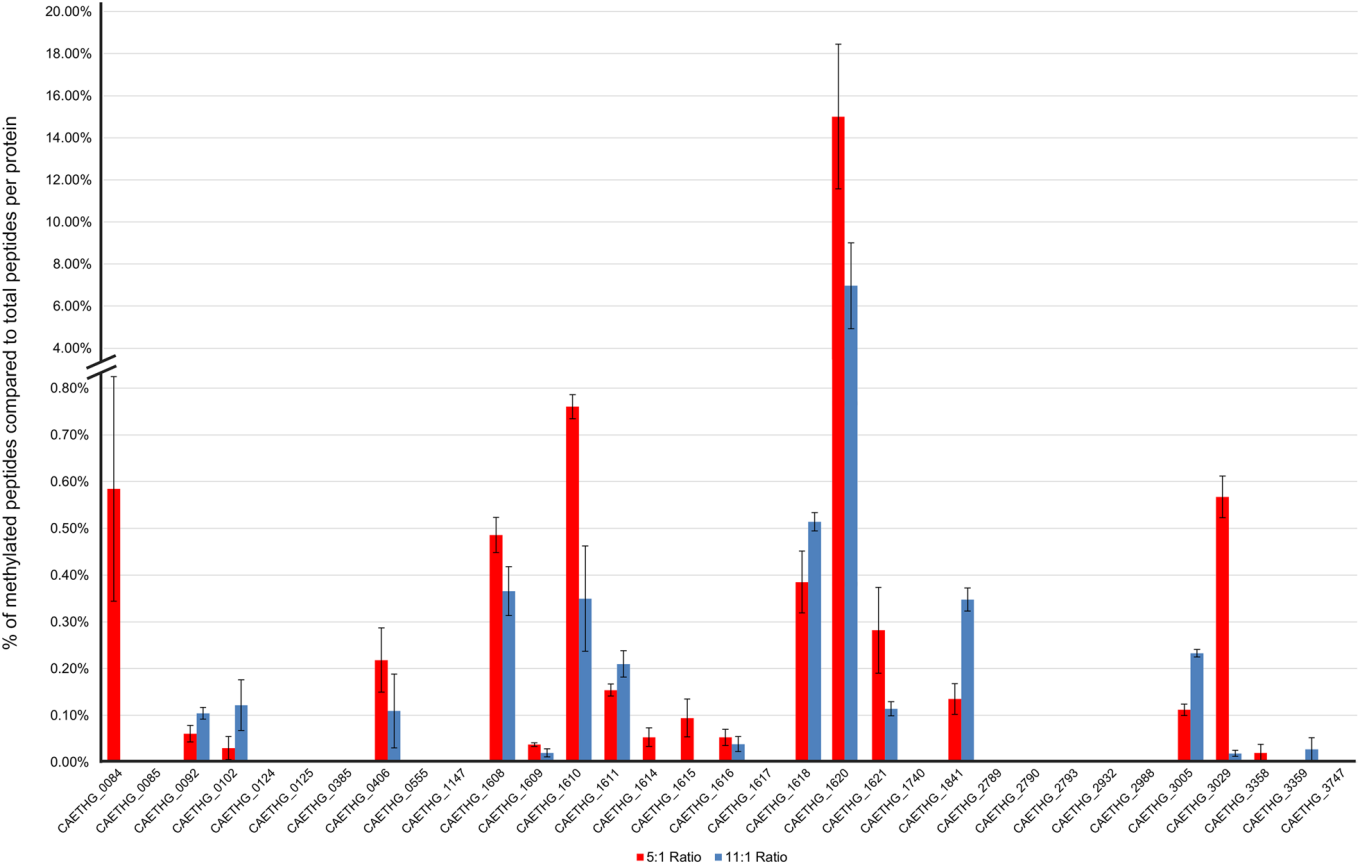
Methylation of WLP proteins. A bar chart depicting the percentage of methylated peptides in proportion to total detected peptides for individual WLP proteins. The red bars represent the 5:1 H_2_:CO ratio condition and the blue the 11:1 condition

Trends of high percentages of methylation were observed on peptides mapping to proteins involved in/related to the WLP, where 19 of the 33 proteins had some form of lysine or arginine methylation (Fig. 4). Of these methylated WLP proteins, the highest methylation percentages were observed in the ACS/CODH complex subunits; many of these proteins showed large abundance shifts in methylation peptides due to condition, however, no over-arching trends of these protein shifts were present. To our knowledge, these results are the first report of methylation in the WLP, and protein specific methylation changes in different gas ratios could indicate the presence of additional regulatory factors in *C. autoethanogenum* that deserve future examination.

### Biotinylation

Recent research has indicated that two key proteins, the biotin-carrier component of acetyl-CoA carboxylase complex (ACC, CAETHG_0127) and pyruvate carboxylase (PYC, CAETHG_1594), are regulated post-translationally by BirA biotinylation in the related bacterium, *C. ljungdahlii* [26]. This report prompted an examination of biotinylation in *C. autoethanogenum*. The search for biotinylation revealed 33 proteins that are biotinylated on a lysine or N-terminus in at least one replicate from either condition or any timepoint. Of these proteins, 29 exhibited N-terminal biotinylation, and 5 had lysine-specific biotinylation (Additional File 5: Table S13). Only one of these proteins showed biotinylation across all conditions, CAETHG_0963, a GDSL esterase/lipid family protein. This constitutive biotinylation of CAETHG_0963 could indicate an elevated importance of this protein in *C. autoethanogenum*, potentially correlated with metabolomics results below reporting high fatty acid accumulation in these high H_2_:CO conditions but additional experiments are needed to confirm the nature of this role.

Regarding ACC and PYC, which are known to be regulated in *C. ljungdahlii* by BirA, both proteins were identified in *C. autoethanogenum* with multiple peptides expressed across conditions, however neither ortholog had a measured biotinylated peptide. This lack of biotinylation may be due to specific conditional differences, lack of PTM enrichment, lysine biotinylation disrupting trypsin cleavage, or lack of conservation of the BirA regulatory function in *C. autoethanogenum*.

In the WLP, CAETHG_1608, the acetyl-CoA synthase, was the only protein that showed evidence of biotinylation. Only one peptide was found with N-terminal and lysine biotinylation in the high H_2_:CO ratio day 7 samples. This low presence of biotinylation likely indicates that biotin may not be the predominant PTM regulating the *C. autoethanogenum* but could still be involved in small fine-tuning regulation in related pathways.

### Metabolomic profiles show distinct differences between conditions and temporally within a condition

Contrasting the metabolomic profiles of the high H_2_:CO ratio cultures that were at steady state (high H_2_:CO ratio day 21 and high H_2_:CO ratio day 14) with those cultures at an earlier time point (high H_2_:CO ratio day 7) indicated that samples from high H_2_:CO ratio day 14 were variable and statistically similar to those of day 7, but those of the later stage (high H_2_:CO ratio day 21) clearly differed from those of the early-stage (high H_2_:CO ratio day 7) (Additional File 6: Table S14). The metabolomic profile of the steady-state of the high H_2_:CO ratio cultures were characterized by accumulations of fatty acids/fatty alcohols, including 1-tetradecanol, tetradecanoic acid (C14:0), cis-10-isoheptadecenoic acid (C17:1), nonanoic acid (C9:0), and dodecanoic acid (C12:0). Also accumulating were fatty acid/lipid-related metabolites, including glycerol-1,3-diphosphate, glycerol 3-phosphate, and one of the glycerol-fatty acid conjugates; 1-myristoleoyl-glycerol. Concurrent with the accumulation of lipid-related metabolites as cultures age was a decline in meso-2,3-butanediol, a fuel molecule (Additional File 6: Table S14). The time-course comparison within the high H_2_:CO ratio condition additionally showed a depletion of nitrogenous metabolites, including the amino acids; alanine, aspartic acid, and phenylalanine, and the vitamin nicotinic acid (niacin; vitamin B3) (Additional File 6: Table S14).

The comparison of the metabolomic profiles of the high H_2_:CO ratio cultures versus the low H_2_:CO ratio cultures under steady-state conditions indicated glycerol-fatty acid conjugates were lower in the high gas ratio cultures, including 1-lauroyl-glycerol, 1-palmitoleoyl-glycerol, 1-myristoyl-glycerol, and 2-monomyristin (Additional file 6: Table S15). Similarly, several phosphorylated intermediates were also lower, including glucose 6-phosphate, glycerol 3-phosphate, and ethyl phosphate. Overall, the metabolomics data suggest the high H_2_:CO ratio treatment supplied the reductant required to limit the accumulation of carbon reserves in the form of glycerol-fatty acid conjugates.

## Discussion

Our investigation into stoichiometric optimization and employment of distinct gas compositions targeting dramatically elevated H_2_:CO uptake ratios demonstrated consistent biomass concentrations and product yield while optimizing CO_2_ fixation. Further, the proteomic and metabolomic analyses in this study shed light on the distinct responses of *C. autoethanogenum* under the two different gas ratio conditions targeting 5:1 and 11:1 H_2_:CO uptake ratios at steady-state growth conditions. The examination of protein abundance changes, post-translational modifications (PTMs), and metabolite profiles provides valuable insights into how the microbial metabolism adapts to variations in the H_2_:CO ratio.

The proteins within the WLP were among the most abundant across the two conditions, indicative of their key role for C1 metabolism and consistent with previous studies [23, 28]. Notably, the WLP exhibited very minor protein abundance changes. In the comparison between the 5:1 and 11:1 condition, differences in protein abundance primarily were observed in vitamin B12 synthesis. The decrease in abundance of vitamin B12 biosynthesis genes—mostly all of which trended similarly—in the high H_2_:CO condition and the adjustments in hydrogen utilization and energy metabolism both reflect the ability of the organism to cope with gas ratio changes via fine-tuning pathways that feed into and affect the WLP. This study corroborates recent results in the closely related organism *C. ljungdahlii*, which has a clear preference for CO but flexibly adapts its metabolism to feedstocks high in H_2_ and low in CO [29].

The consistency of WLP protein abundances suggests that additional regulatory mechanisms, like post-translational modification, might be deployed to adapt to changing gas ratios. Indeed, the identification of a significantly differentially expressed acetyltransferase emphasizes the potential importance of acetylation in *C. autoethanogenum* metabolic pathway regulation. Coupling this observation to previous studies into PTMs of WLP enzymes in a closely related acetogen led to the investigation of the PTMs acetylation, methylation, and biotinylation under CO_2_ fixation conditions in this study [24, 26]. Lysine-acetylation profiles highlight pathways affected by this modification, mainly the WLP and end-product fermentation. The exploration of protein methylation and biotinylation adds another layer to the regulatory landscape. High incidences of methylation in WLP proteins, especially in the CODH/ACS complex, suggest that PTMs act as potential regulatory factors affecting gas uptake and bio-product formation in this acetogenic microbial system. The distribution of increased acetylation and methylation within distinct segments of the WLP raises the possibility of channeling product flux through PTM-mediated mechanisms, offering a more detailed perspective on the intricate regulation of carbon fixation in the WLP. Biotinylation regulates central metabolism protein activity in *C. ljungdahlii* [26], and even though biotinylation in this study exhibited decreased prevalence, it could still be important for key proteins. Regardless, the prevalence and flux of these PTMs between conditions point to a crucial need to study the role of PTMs under different physiological conditions and potential regulation in *C. autoethanogenum*.

The metabolomic profiles provide additional context, revealing differences between the 5:1 and 11:1 conditions in terms of glycerol-fatty acid conjugates and phosphorylated intermediates. These changes indicate variations in carbon and nitrogenous metabolites, emphasizing the impact of gas ratio on the microbial metabolic response. The time-course comparison of the high H_2_:CO ratio cultures indicated the accumulation of the fatty acid precursors, but without the broad-based accumulation of the fatty acid-glycerol conjugates.

## Conclusions

In total, this work reveals molecular details of the interplay of multiple factors influencing carbon fixation in *C. autoethanogenum* under varying H_2_:CO ratios, including the previously uncharacterized 11:1 ratio which is able to achieve 75% direct CO_2_ fixation to ethanol. The manipulation of gas feed composition was demonstrated to significantly impact carbon conversion and product yield, emphasizing the importance of optimal H_2_:CO ratios for carbon efficiency. While proteomic profiling revealed an invariance of foundational proteins within the Wood–Ljungdahl pathway, these measurements provided the first experimental evidence that fine-tuning enzymatic activities via post-translational modifications likely contribute to regulation across conditions. Notably, the prominence of acetylation and methylation, which is reported here for the first time in acetogens during gas fermentation, within the WLP suggests a novel flux optimizing mechanism for carbon fixation. The correlation between redox control and product flux also highlights a potential avenue for enhancing desired product yields. Metabolomics also revealed the carbon storage shifts impacted by H_2_:CO ratios. Overall, this comprehensive study provides previously unknown insights into the intricate orchestration of factors impacting carbon fixation and conversion at ultra-elevated H_2_:CO ratios and sets the stage for further research to decipher the functional significance of individual components that enable industrial bioprocess optimization.

## Materials and methods

### Strains and chemicals

*C. autoethanogenum* DSM 19630, a derivate of the type strain DSM10061 was used for all studies. Cultures were handled using anaerobic techniques and medium were described previously [19, 20]. Molecular-grade chemicals (Sigma-Aldrich, Inc., St. Louis, MO) and reverse-osmosis water were used for laboratory experiments.

### Fermentation and end-point quantification

Continuous fermentations were carried out under strictly anaerobic conditions at 37 °C with constant medium and gas supply as previously described except that 1 L Multifors bioreactors (Infors HT USA, Annapolis Junction, MD) were used in this study [18–20]. Briefly, synthetic gas blends composing 10:1 (50% H_2_, 5% CO) or 20:1 H_2_:CO (60%H_2_, 3%CO) were fed to target 5:1 (low) or 11:1 (high) H_2_:CO uptake ratios, respectively, with 30% CO_2_ and balance N_2_ (AirGas, Radnor Township, PA) at atmospheric pressure. Liquid dilution rates were targeting 0.9–1.0 day^−1^ and gas flow rates targeted 100 normal cubic centimeters per minute (NCCM) per liter. Gas flow rates in and out of vessels were quantified with mass-flow controllers (Alicat Scientific, Tuscon, AZ).

Gas and metabolite measurements were automatically sampled every 1–3 h as described previously [19]. Biomass samples were taken 1–2 times per day and concentrations were determined using a spectrophotometer as described previously [19]. Metabolites, ethanol, acetic acid, lactic acid, and 2,3-butanediol were measured from fermentation broth using an Agilent 1100 or 1200 series HPLC as described previously [19, 30]. Headspace gas composition data were measured by gas chromatography using a 490 Micro GC (Agilent Technologies, Santa Clara, CA) as previously described [19]. For productivity and selectivity calculations, only concentrations in the broth were considered (i.e., not considering any stripped product). Carbon monoxide or hydrogen utilization was calculated as follows: %Gas Consumed = ((% Gas_IN_ * Outlet flow) − (%Gas_IN_ * Inlet flow))/(%Gas_IN_ * Inlet flow).

### LC–MS/MS proteomic sample preparation and data analysis

Samples (in biological triplicates) were harvested from the high H_2_:CO ratio condition at days 7, 14, and 21, as well as from the low H_2_:CO condition at days 20 and 43 and frozen at − 80 °C until proteomic analysis. For cell pellet lysis, pellets were thawed on iced then re-suspended in 400 µL of Tris buffer (Tris–HCl, Tris Base, and H_2_O; 100 mM at a pH of 8.0) and added on top of 0.15 mm beads (~ 200 μl). Samples were then bead-beat using the Cryogrinder at 1750 rpm for 5 min to penetrate the cell membranes. Lysis buffer was added to samples to a final concentration of 4% sodium dodecyl sulfate and 10 mM Dithiothreitol. The samples were then heated at 90 °C for 10 min with constant shaking in order to denature proteins and reduce disulfide linkages. Once lysed, a NanoDrop OneC Microvolume UV–vis spectrophotometer (Thermo Scientific) was used to determine the crude protein amount by measuring the corrected absorbance at 205 nm. Once quantified, crude proteins were alkylated with 30 mM iodoacetamide and incubated in the dark for 15 min to prevent the reformation of disulfide bonds. Approximately, 300 μg of protein for each sample was used for protein aggregation capture (PAC) [31] and subsequent trypsin digestion. Briefly, a 1:1 ratio of magnetic beads (1 μm SpeedBead magnetic carboxylate modified particles; GE healthcare UK) was suspended in each sample. Acetonitrile (ACN) was added to each sample to reach a final concentration of 70%. Samples were vortexed, allowed to settle for 10 min, vortexed and allow to settle for a final 10 min. This allowed the proteins to aggregate to the beads so the crude protein could be cleaned before digestion. Samples were placed on a magnetic rack. Samples were washed with 1 mL of ACN and 1 mL 70% ethanol. Samples were removed from the magnetic rack and 100 mM ammonium bicarbonate was added to resuspend the beads. A 1:75 ratio of MS grade trypsin protease (Pierce-Thermo Scientific) in trypsin buffer (30 mM Acetic acid in MS grade H_2_O) was added for peptide digestion for 3 h at 37 °C and then again overnight. A final concentration of 0.5% formic acid (FA) was added to acidify peptides. Finally, supernatant containing tryptic peptides was filtered through 10 kDA MWCO centrifugal concentrator (Vivaspin500 PES; Sartorius) in order to remove under-digested proteins. Final quantification of resulting peptides was measured with NanoDrop OneC.

A Vanquish UHPLC system coupled to an Orbitrap Q-Exactive Plus mass spectrometer (Thermo Scientific) and reverse-phase 1D LC–MS/MS was used to analyze 3 μg of peptides from each sample. Peptides were trapped on a single frit trapping column (100 μm ID) packed with 6 cm of C18 resin (5 μm Kinetex; Phenomenex) and separated by an organic gradient on a nanospray emitter (75 μm ID) packed with 15 cm of C18 resin (1.7 μm Kinetex; Phenomenex). This sample loading, trapping, then desalting went on for 30 min at 2 μL/min in solvent A [0.1% FA in 5% acetonitrile (ACN)]. A split flow rate at 300 nL/min was used for analytical separation and peptide elution with a gradient that started at 0–30% solvent B (0.1% FA in 70% ACN) over 185 min then increased to 100% solvent B over 5 min, until holding at 100% solvent for 15 min. A column wash followed each elution. The orbitrap Q-Exactive Plus mass spectrometer was set in data- dependent mode and measured and sequenced eluting peptides. Full-scan MS spectra were collected during the first 240 min of the 275 min total analysis time and measured in the range m/z 300–1500 at a resolution of 70,000 (full width at half maximum), with an AGC target value of 1 × 10^6^. At a normalized collision energy of 27%, AGC target value of 1 × 10^6^, and resolution of 17,500 higher energy collisional dissociation fragmentation was performed. For MS/MS, 20 of the most intense precursor ions were selected with an isolation window set to 1.8 m/z and a 30 s dynamic exclusion window.

The resulting peptide fragmentation was analyzed using the MS Amanda algorithm (v2.0) in Proteome Discoverer software (version 2.3.0.523, Thermo Scientific) using a target decoy approach against the *C. autoethanogenum* DSM 10061 Uniprot proteome appended to the common protein contaminants database [32]. Peptide requirements were as follows: fully tryptic, two missed cleavages maximum, and five amino acid length minimum. A match between runs was done during a grouped consensus step on triplicate sets, and peptides were then filtered based on a false discovery rate (FDR) scoring of 1%. The methionine residues were oxidized with a dynamic modification of 15.9949 Da and the cysteine residues were carbamidomethylated with a static modification of 57.0214 Da. Protein quantification was calculated by summing peptide abundances from extracted ion chromatograms. Using an R-script, proteins abundances were normalized (log2 transformation followed by loess normalization and median-centering) and missing values were imputed to simulate the MS limit of detection if a protein was present in at least one sample in a triplicate set [33].

Two time points across the chemostats during steady state were used for each condition in the binary comparisons, as this increased the statistical power by increasing the number of replicates used and minimized the possibility of differential protein significance due to stochasticity of protein abundance across chemostats. Within the Perseus interface, two-tailed Student’s *t*-tests were used to measure significant differences in protein abundances between the high H_2_:CO ratio samples and the low H_2_:CO ratio samples at a Benjamini–Hochberg FDR corrected *p* value of ≤ 0.05 [34]. An ANOVA with the same multiple test correction parameters was done for a linear comparison of the high H_2_:CO ratio condition. Proteins with significant differential expression between conditions had *p* values < 0.05 and |log2 Fold Change|> 1. For all comparisons, annotations were downloaded from UniProtKD, KEGG, and EGGNOG [35–37]. The significant proteins were hierarchically clustered using Euclidean distance. Figures displaying proteomic results were created using BioRender.com.

For PTM analysis, separate Proteome Discoverer searches were done with the same settings listed above. A static lysine-acetylation modification, lysine and arginine mono, di, and tri-methylation, or lysine biotinylation filter was added. Missed cleavages were increased to 3 for biotinylation. Peptide filtering and protein-roll up also followed previously stated protocol. Normalization was done as described above and then normalized peptide abundances were taken out of log2 space for percentage roll-ups and analysis. Percentage abundances were calculated by dividing the abundance of modified peptides by the total peptide abundance for the protein.

### GC–MS metabolomic sample preparation and data analysis

Samples (in biological triplicates) for metabolomic analysis were similarly harvested and stored at − 80 °C as described above for LC–MS/MS. For metabolite extraction, frozen cell pellets were weighed into centrifuge tubes containing 5 mL of 80% ethanol to which 40 μL of sorbitol (1 mg*mL-1 aq) had been added as internal standard. Samples were sonicated (Q500 Sonicator, Qsonica Sonicators) for 3 min at 30% amplitude (30-s on-and-off cycles) while kept chilled in an aluminum cooling block, followed by centrifugation at 4200 rpm for 20 min at 4 °C. The supernatant was decanted and a 2 mL aliquot was dried under a stream of nitrogen, dissolved in 0.5 mL silylation grade acetonitrile (TS-20062, Thermo Scientific), followed by addition of 0.5 mL of 2,2,2-Trifluoro-N-methyl-N-(trimethylsilyl)-acetamide plus 1% Chlorotrimethylsilane (MSTFA plus 1% TMCS, Thermo Scientific) and heated for 1 h at 70 °C to generate trimethylsilyl derivatives. An Agilent 7890A gas chromatograph coupled to a 5975C inert XL mass spectrometer (GC–MS) was used to analyze 1μL aliquots 2 days post-derivatization. The GC was fitted with an RTX-5MS with 5 m Integra-Guard (5% diphenyl/95% dimethyl polysiloxane, 30 m × 0.25 mm ID, 0.25 μm film thickness) capillary column and the GC was configured using parameters previously described [38], and a gas (helium) flow of 1.2 mL per min. The MS was operated in electron ionization mode (70 eV) full-spectrum scan (50–650 m/z mass range) with parameters also previously described [38]. Metabolites peaks were extracted using a unique, characteristic m/z fragment to minimize integration of co-eluting metabolites, and for known metabolites, scaled back to total ion current (TIC) using previously calculated TIC/(m/z) scaling factors. Peaks were quantified by area integration and normalized to the internal standard recovered, sample mass, extract fraction analyzed, derivatization volume, and volume injected. Peak assignment was made using a Wiley Registry 12th Edition/NIST 2020 Mass Spectral Library and a large user-created database (> 2700 signatures). For unidentified metabolites, the scaling factor for the internal standard was used, and these metabolites were reported based on their retention times (RT; min) and key m/z. The statistical treatment of the metabolite data was as follows: for sample set 1, the mean metabolite concentrations of pellet samples for 11:1 ratio day 14 and day 21 were compared to those of 11:1 ratio day 7 using Student’s *t*-tests. For sample set 2, the mean metabolite concentrations of pellet samples for cultured under the 11:1 ratio (Day 14 + 21) were contrasted with those cultured under the 5:1 ratio (Day 20 + 43). For all contrasts, treatment means were significantly different at *p* value < 0.05.

### Supplementary Information

**Additional file 1:** Supplemental Tables 1–5. Proteome Discoverer Results **Table S1.** All Conditions Protein results. Proteome Discoverer protein results filtered for common contaminates. **Table S2**. Peptide results. Proteome Discoverer peptide results filtered for common contaminates. **Table S3.** Peptide results with Acetylation Modifications. Proteome Discoverer peptide results with added search parameter to look for lysine-acetylation and filtered for common contaminates. **Table S4.** Peptide results with Biotinylation Modifications. Proteome Discoverer peptide results with added search parameter to look for lysine biotinylation and filtered for common contaminates. **Table S5.** Peptide results with Methylation Modifications. Proteome Discoverer peptide results with added search parameter to look for single, double, or triple methylation on Lysine or arginine residues and filtered for common contaminates. NA = not detected.

**Additional file 2:** Supplemental Figures 1–3. **Figure S1.** Separation of Proteomes by Condition. A PCA plot showing the groupings of replicates for each of the conditions. **Figure S2.** Total Lysine Acetylation Percentage. A bar chart depicting the percentage of acetylated peptides in proportion to total detected peptides. **Figure S3.** The Total Lysine and Arginine Methylation Percentage. A bar chart depicting the percentage of methylated peptides in proportion to total detected peptides. The red represents peptides methylated on the arginine residues, and the blue peptides methylated at lysine residues.

**Additional file 3:** Supplemental Tables S6-9. 11:1 H_2_:CO Condition Linear Comparison Quantitative proteomic analysis Results. **Table S6.** Anova Test for 11:1 Condition Timecourse. The abundances shown (in Day# columns along with hidden replicate columns) are normalized, log transformed, and imputed. Protein abundance numbers shown in red indicates imputed values. **Table S7**—11:1 Condition Day 7 vs Day 21 Ttest results. In the Log2FoldChange column are highlighted green if it favors day 7 and red if it favors day 21 bolded numbers have a value >|1|. The Significant column indicates protein with a *p*-value >0.05 and fold change <|1|. Protein abundance numbers shown in red indicates imputed values. **Table S8**—11:1 Condition Day 7 vs Day 14 Ttest results. In the Log2FoldChange column are highlighted green if it favors day 7 and red if it favors day 14 bolded numbers have a value >|1|. The Significant column indicates protein with a *p*-value >0.05 and fold change <|1|. Protein abundance numbers shown in red indicates imputed values. **Table S9**—11:1 Condition Day 14 vs Day 21 Ttest results. In the Log2FoldChange column are highlighted green if it favors day 14 and red if it favors day 21 bolded numbers have a value >|1|. The Significant column indicates protein with a *p*-value >0.05 and fold change <|1|. Protein abundance numbers shown in red indicates imputed values.

**Additional file 4:** Supplemental Table S10. Binary Comparison Quantitative proteomic analysis Results. **Table S10.** Low vs High H2:CO Condition Ttest Comparison. The abundances shown (in 5:1 uptake and 11:1 uptake columns along with hidden replicate columns) are normalized, log transformed, and imputed. Protein abundance numbers shown in red indicate imputed values. T-tests compare the Day 43 and 20 samples from the 5:1 H_2_O condition to the Day 14 and day 21 samples from the 11:1 H_2_O condition.

**Additional file 5:** Supplemental Tables S11-13. PTM Results (Acetylation, Methylation, and Biotinylation). **Table S11.** Protein Lysine Acetylation Percentage (acetylated peptide abundance/total peptide abundance*100). NF = no peptides found. 0 = non-acetylated peptides are present but no acetylated. **Table S12.** Protein Lysine &/or Arginine Methylation Percentage (Lysine or Arginine Methylated peptides abundance/total peptide abundance*100). NF = no peptides found. 0 = non-Methylated peptides are present but no Methylated. **Table S13.** Protein Lysine or N-terminal Biotinylation Percentage (biotinylated peptide abundance/total peptide abundance*100). NF = no peptides found. 0 = non-biotinylated peptides are present but no biotinylated

**Additional file 6:** Supplemental Tables S9-10. Metabolomics Results. **Table S14.** 11:1 H2:CO Condition Metabolomics Results. Metabolite values highlighted in red indicate a *p*-value < 0.05. **Table S15.** 5:1 vs 11:1 Metabolomic Results. Metabolite values highlighted in red indicate a *p*-value < 0.05.

## Data Availability

All data for this manuscript, including the global proteome results of each sample, are included as additional files. *C. autoethanogenum* DSM10061 are deposited at the German Collection of Microorganisms and Cell Cultures GmbH (DSMZ). The reference genome used in this study is available from the National Center of Biotechnology Information GenBank under accession CP006763.1.

## Abbreviations

2,3-BDH: 2,3-Butanediol dehydrogenase
ACC: Acetyl-CoA carboxylase
Ack: Acetatekinase
ACN: Acetonitrile
ACS: Acetyl-CoA synthase
Adh/AdhE: Alcohol dehydrogenase
AlsS: Acetolactate synthase catabolic
AOR: Acetaldehyde:ferredoxin oxidoreductase
BudA: Acetolactate decarboxylase
CO: Carbon monoxide
CODH: CO dehydrogenase
CTSR: Continuous stirred tank reactor
FA: Formic acid
Fdh: Formate dehydrogenase
FDR: False-discovery rate
FTHFS: Formyltetrahydrofolate synthetase
Hyt: Electron-bifurcating NADP- and ferredoxin-dependent hydrogenase
LdhA: Lactate dehydrogenase
MTHFC: Methenyltetrahydrofolate cyclohydrolase
MTHFD: Methenyltetrahydrofolate dehydrogenase
MTHFR: Methylenetetrahydrofolate reductase
NFN: NADH-dependent reduced ferredoxin:NADP + oxidoreductase
PFOR: Pyruvate:ferredoxin oxidoreductase
Pta: Phosphotransacetylase
PTM: Post-translation modifications
PYC: Pyruvate carboxylase
Redox: Reduction–oxidation
Rex: Redox regulator
RNF: Membrane-associated and energy-conserving reduced ferredoxin:NAD + oxidoreductase
TIC: Total ion current
WLP: Wood–Ljungdahl pathway
