# Supplementary material for: Clostridium autoethanogenum alters cofactor synthesis, redox metabolism, and lysine-acetylation in response to elevated H2:CO feedstock ratios for enhancing carbon capture efficiency
Source: Biotechnol Biofuels Bioprod. 2024 Sep 3;17:119. doi: 10.1186/s13068-024-02554-w (PMC11370222; doi:10.1186/s13068-024-02554-w)
Supplement: Supplementary file 2 — Additional file 2: Supplemental Figures 1–3. Figure S1. Separation of Proteomes by Condition. A PCA plot showing the groupings of replicates for each of the conditions. Figure S2. Total Lysine Acetylation Percentage. A bar chart depicting the percentage of acetylated peptides in proportion to total detected peptides. Figure S3. The Total Lysine and Arginine Methylation Percentage. A bar chart depicting the percentage of methylated peptides in proportion to total detected peptides. The red represents peptides methylated on the arginine residues, and the blue peptides methylated at lysine residues. [file 13068_2024_2554_MOESM2_ESM.docx]

**Supplementary Tables & Figures**


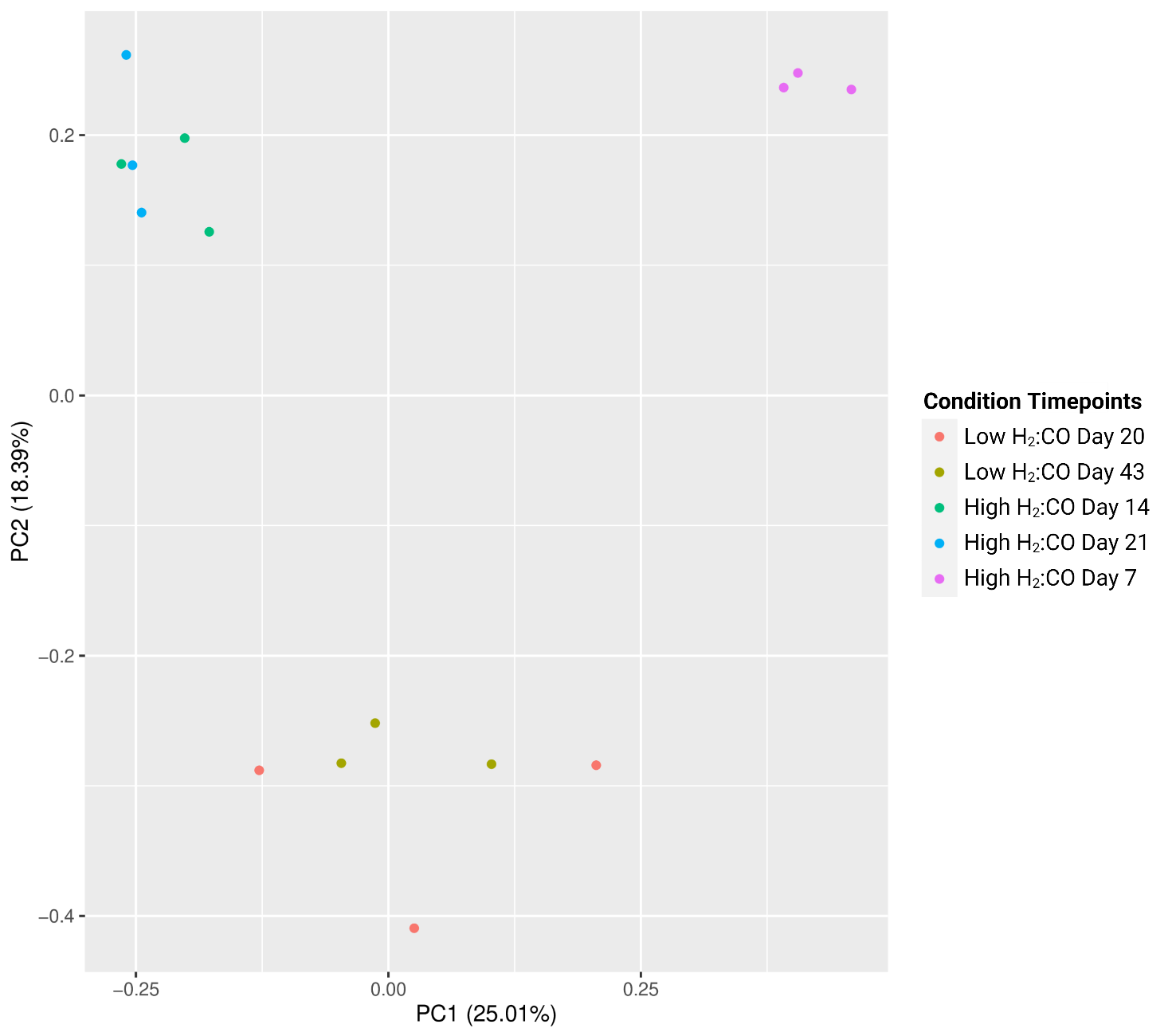
**Figure S1. Separation of Proteomes by Condition.** A PCA plot showing the groupings of replicates for each of the conditions.


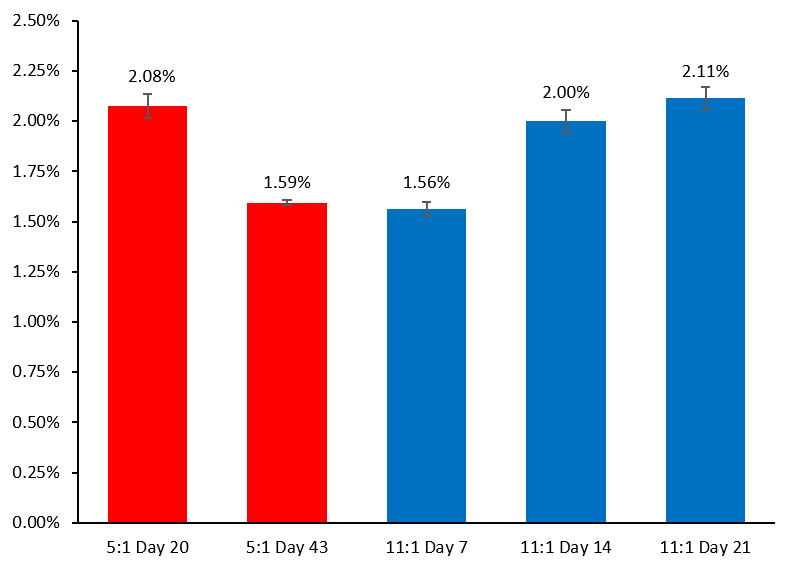


**Figure S2. Total Lysine Acetylation Percentage.** A bar chart depicting the percentage of acetylated peptides in proportion to total detected peptides.


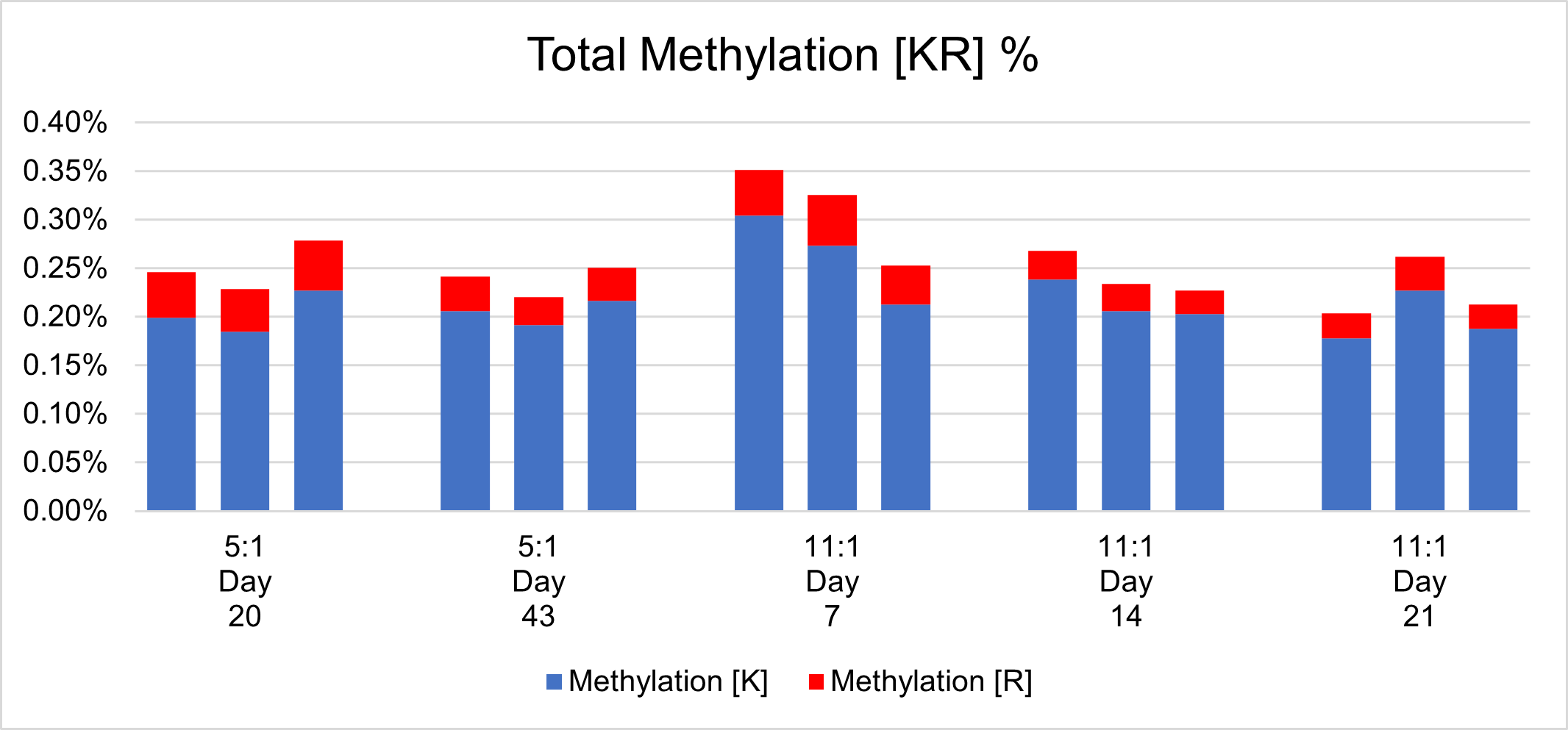


**Figure S3. The Total Lysine and Arginine Methylation Percentage.** A bar chart depicting the percentage of methylated peptides in proportion to total detected peptides. The red represents peptides methylated on the arginine residues, and the blue peptides methylated at lysine residues.
